# Reply: Time‐Related Biases in Observational Studies of GLP‐1 Agonists and Cancer Risk

**DOI:** 10.1111/1753-0407.70203

**Published:** 2026-03-08

**Authors:** Zhiyuan Cheng, Jiesheng Lin, Dongfeng Gu, Fengchao Liang

**Affiliations:** ^1^ School of Public Health and Emergency Management, School of Medicine Southern University of Science and Technology Shenzhen China; ^2^ Department of Epidemiology School of Public Health, Sun Yat‐sen University (North Campus) Guangzhou Guangdong China

We thank the authors of the letter for their thoughtful comments on our work [1] and appreciate the opportunity to clarify our methodological choices.

Our real‐world, population‐based study suggests an association between GLP‐1 receptor agonist (GLP‐1RA) use and increased risks of adverse outcomes like pancreatitis, kidney issues, and thyroid disorders [2]. Regarding exposure definition, the correspondents correctly note that we adopted a predominant‐user rather than a strict incident new‐user design. New‐user designs are preferred for single‐outcome causal inference, particularly for cancer. However, its feasibility is constrained by real‐world treatment patterns and data availability in multi‐outcome safety evaluations. In China, GLP‐1RAs are typically prescribed as add‐on third‐line (2010–2017) or second‐line (2017–2024) treatment for type 2 diabetes. Requiring patients to be free of all antidiabetic medications at cohort entry would exclude a large proportion of GLP‐1RA users and compromise representativeness. In addition, prescription data before 2016 were unavailable in Shenzhen health information systems, limiting reliable identification of true incident users. Under these conditions at the time of publication, a strict new‐user design was not practically feasible, although such designs may become increasingly viable as data accrual and follow‐up continue. Notably, several large observational studies of GLP‐1RA safety similarly did not apply a pre‐baseline washout period, reflecting comparable real‐world considerations [1, 4]. As real‐world experience with GLP‐1RAs accumulates, further research specifically focused on the initiation of therapy remains warranted.

We acknowledge that the absence of a washout requirement to exclude pre‐existing or early‐diagnosed cancers could indeed introduce prevalent‐user bias, which is a valid methodological concern. However, in unsupervised real‐world settings, treatment interruption and re‐initiation are common and are largely driven by tolerance and adherence. Thus, imposing a washout period under these conditions may introduce additional selection bias and further reduce statistical power, particularly for uncommon outcomes. As data coverage expands with longer follow‐up, alternative exposure definitions merit further evaluation.

A lag period appropriately addresses detection bias, which is especially relevant for cancer outcomes where early diagnosis might coincide with treatment initiation rather than being caused by the treatment itself. However, our study assessed a broad spectrum of 20 adverse outcomes, with latencies ranging from short‐term (≤ 12 months) to long‐term (> 12 months), as part of a comprehensive safety evaluation. We rechecked our data on incident cases of thyroid cancer, and only 11.5% (3/26) of incidents in the GLP‐1RA group occurred under 12 months of follow‐up, compared with 33.3% (8/24) in the metformin group. Thus, these data may argue against this bias being a primary driver of the observed association. Moreover, given the relatively short follow‐up and limited number of cancer events, introducing a lag period would have further compromised statistical power. We therefore used duration‐stratified analyses to explore temporal patterns while preserving sensitivity to early‐onset adverse effects.

We are collaborating with municipal and provincial health information databases in China. Future analyses will be able to incorporate additional methodological refinements, including alternative exposure definitions and lagged designs as suggested, to further enhance the robustness of the evidence.

## Author Contributions

Design of the work and analysis: Zhiyuan Cheng. Draft and review: Zhiyuan Cheng, Jiesheng Lin, Dongfeng Gu, and Fengchao Liang. Final approvement: Dongfeng Gu and Fengchao Liang.

## Funding

The authors have nothing to report.

## Conflicts of Interest

The authors declare no conflicts of interest.

## Data Availability

Data sharing not applicable to this article as no datasets were generated or analysed during the current study.
